# Supplementary material for: Topologically driven linear magnetoresistance in helimagnetic FeP
Source: npj Quantum Inf. Author manuscript; Available in PMC 2023 Sep 20. (PMC10510734; doi:10.1038/s41535-021-00337-2)
Supplement: Supp1 [file NIHMS1918184-supplement-Supp1.pdf]

Supplementary Information: Topologically-Driven Linear Magnetoresistance in Helimagnetic FeP

D.J. Campbell,[1,*] J. Collini,[1,2] J. Sławińska,[3,†] C. Autieri,[4,5] L. Wang,[1] K. Wang,[1] B. Wilfong,[1,6] Y.S. Eo,[1] P. Neves,[1,2] D. Graf,[7] E.E. Rodriguez,[1,6] N.P. Butch,[1,2] M. Buongiorno Nardelli,[3] and J. Paglione[1,8,‡]

[1]*Maryland Quantum Materials Center, Department of Physics, University of Maryland, College Park, Maryland 20742, USA*
[2]*NIST Center for Neutron Research, NIST, Gaithersburg, Maryland 20899, USA*
[3]*Department of Physics, University of North Texas, Denton, TX 76203, USA*
[4]*International Research Centre Magtop, Institute of Physics, Polish Academy of Sciences, Aleja Lotników 32/46, PL-02668 Warsaw, Poland*
[5]*Consiglio Nazionale delle Ricerche CNR-SPIN, UOS Salerno, I-84084 Fisciano (Salerno), Italy*
[6]*Department of Chemistry, University of Maryland, College Park, Maryland 20742, USA*
[7]*National High Magnetic Field Laboratory, 1800 East Paul Dirac Drive, Tallahassee, Florida 32310, USA*
[8]*Canadian Institute for Advanced Research, Toronto, Ontario M5G 1Z8, Canada*
(Dated: March 22, 2021)

* daniel.campbell@lncmi.cnrs.fr, Present address: LNCMI, CNRS, EMFL, Univ. Grenoble Alpes, INSA Toulouse, Univ. Toulouse Paul Sabatier, 38000 Grenoble, France
† Present address: Zernike Institute for Advanced Materials, University of Groningen, Nijenborgh 4, 9747AG, Netherlands
‡ paglione@umd.edu

SUPPLEMENTARY NOTE 1. COBALT PHOSPHIDE

We grew crystals of cobalt phosphide (CoP), with a similar iodine CVT technique to the one used for FeP (see Methods in main text). In contrast to FeP, we noticed CoP grew equally well from the elements or prereacted CoP powder. Like other B31 phosphides, CoP has a similar room temperature resistivity and temperature dependence to its corresponding arsenide (Supp. Ref. [1]), but a much lower residual resistivity [Supplementary Figure 1(a)]. A selection of high field magnetoresistance curves at various angles in the a-c plane are shown in Supplementary Figure 1(b). The magnetoresistance is sizable, though still about an order of magnitude smaller than in FeP. All curves become linear at high field, with minimum MR near $\mathbf{B} \parallel c$-axis. None showed the same low field linear behavior, though the $\mathbf{B} \parallel c$-axis data did have the subtlest curvature. Another difference from FeP is that maximum MR comes for field along the [101] direction, rather than the a-axis.

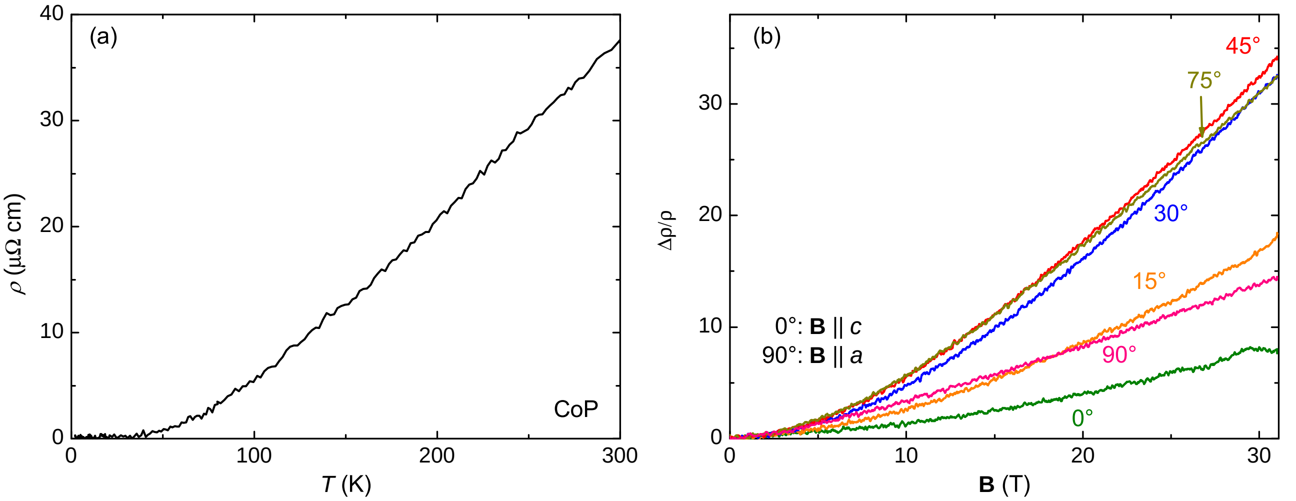

Supplementary Figure 1. (a) Resistivity as a function of temperature for a CoP single crystal. The residual resistivity is about 0.1 $\mu\Omega$ cm. (b) Magnetoresistance data for CoP taken at the NHMFL for field rotated in the a-c plane.

SUPPLEMENTARY NOTE 2. MAGNETORESISTANCE OF DIFFERENT PNICTIDE BINARIES

As noted in the main text, other binary pnictide systems, whether they share the MnP-type *Pnma* structure or not, have shown large magnetoresistance and generally interesting behavior in large fields. Here we compare a few of these systems to put into perspective what we are reporting on FeP.

Supplementary Table I. A comparison of the maximum magnetoresistance observed for several pnictide compounds with a compositional or structural link to FeP. Data are listed for the conditions with the highest MR, except for FeP where list both the highest and linear MR. n refers to the exponent describing the approximate power law dependence of the MR. The listing "1-2" denotes materials that start out initially quadratic that have a linear crossover at higher field (i.e., several Tesla). Note that while the *Pnma* space group is orthorhombic, $I4_1md$ is tetragonal and $C12/m1$ monoclinic.

Compound	Space group	$\mathbf{B} \parallel [hkl]$	MR	$\mathbf{B}$ (T)	T (K)	n
FeP (this work)	*Pnma*	[001]	53	31	0.4	1
FeP (this work)	*Pnma*	[100]	280	31	0.4	2
FeAs (Supp. Ref. [2])	*Pnma*	[100]	1.4	31	0.5	1-2
CoP (this work)	*Pnma*	[101]	34	31	0.6	1-2
CrP (Supp. Ref. [3])	*Pnma*	[001]	360	58	4	2
CrAs (0 GPa) (Supp. Ref. [4])	*Pnma*	[001]	0.6	14	0.026	< 1
CrAs (0.92 GPa) (Supp. Ref. [4])	*Pnma*	[001]	1.2	14	0.016	1
MnP (Supp. Ref. [5])	*Pnma*	[011]	38	8	1.5	2
TaP (Supp. Ref. [6])	$I4_1md$	[001]	300	9	2	1-2
TaAs (Supp. Ref. [7])	$I4_1md$	[001]	800	9	1.8	1
NbP (Supp. Ref. [8])	$I4_1md$	[001]	35000	31	1.3	< 1
NbAs (Supp. Ref. [9])	$I4_1md$	[001]	2500	9	2	< 1
$TaAs_2$ (Supp. Ref. [10])	$C12/m1$	[001]	1000	14	2.5	2
$NbAs_2$ (Supp. Ref. [10])	$C12/m1$	[001]	7300	14	2.5	2

SUPPLEMENTARY NOTE 3. KOHLER SCALING

Supplementary Figure 2(a) shows the magnetoresistance at several different temperatures in the range of 2-50 K for magnetic field aligned along the c-axis of FeP sample S1, the same one presented in the main text. The inset more clearly shows the two highest temperatures, demonstrating while MR magnitude decreases, it still shows linear behavior up to 50 K. Kohler's rule states that the magnetoresistance should be a function of B/ρ_0, where ρ_0 is the resistivity in the absence of field. Thus MR at different temperatures (where ρ_0 is different) should all collapse onto the same curve if the field is divided by zero-field resistivity. Such scaling seems to hold reasonably well for this orientation [Supplementary Figure 2(b)], though it is more difficult to tell at higher temperature due to the very limited B/ρ_0 range. At the very least, Kohler's rule is obeyed more strictly here than for the 5° orientation shown in Fig. 4(a) of the main text.

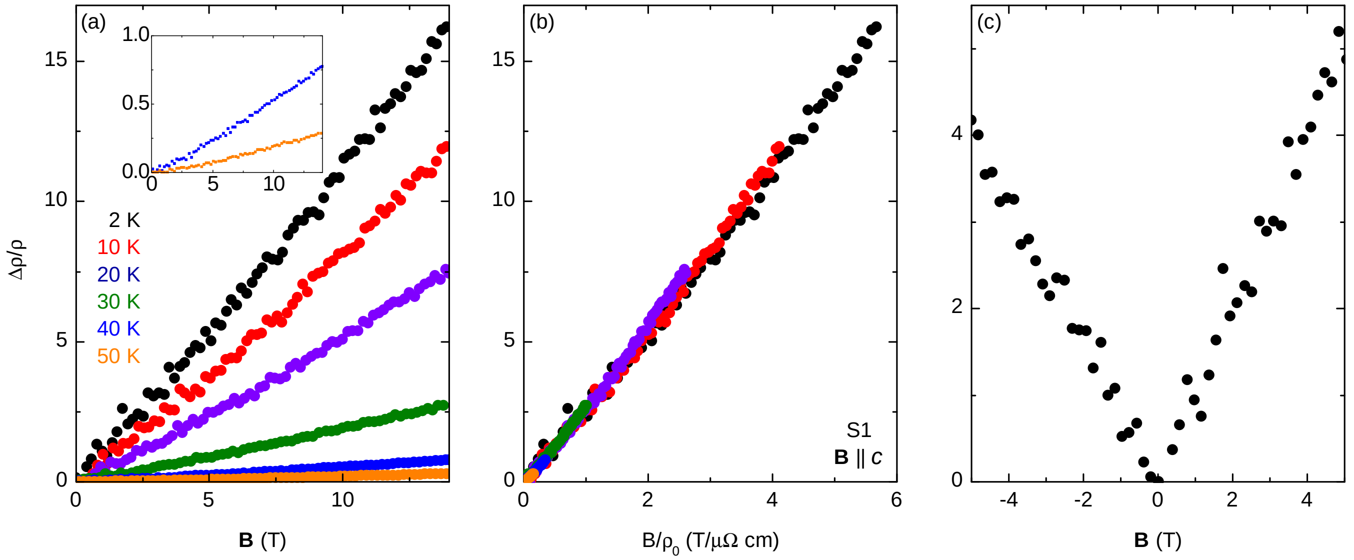

Supplementary Figure 2. Magnetoresistance data and Kohler analysis for FeP sample S1 with field applied **B** ∥ c. (a) Magnetoresistance up to 14 T at various temperatures, showing that linear MR survives up through at least T*. (inset) A zoom-in of the data for 40 K and 50 K, demonstrating that even when MR is much smaller it retains its primarily linear character. (b) The same data as a function of B/ρ_0. For the most part, the curves fall on top of each other, indicating that Kohler's rule is observed at this angle above and below T*. (c) The 2 K data for the same sample between -5 T and +5 T. Linear MR is maintained through 0 T to within resolution of point spacing and variation in the data (the latter a result of the low resistivity). A difference in MR at 5 T due to a parasitic Hall component is also visible.

SUPPLEMENTARY NOTE 4. HALL EFFECT

The Hall resistance was measured for three high RRR samples with different orientations [Supplementary Figure 3(a)], two of which are featured in the main text: **B** ∥ [101] (S1), [010] (S2), and [001] (here called S4). The Hall coefficient R_{H} was extracted by antisymmetrizing ±14 T field sweeps. In FeAs, $R_{\mathrm{H}}(T)$ can change sign multiple times (Supp. Ref. [11]), but in the case of FeP it was negative for all orientations and temperatures. It does have, however, a noticeable temperature dependence, with local extrema (though the wells are very shallow) in the **B** ∥ [001] and [101] samples in the vicinity of T_{N}, and $|R_{\mathrm{H}}|$ minima in all three samples at lower temperatures between 25-75 K. The Hall resistivity ρ_{xy} of Sample S1 (**B** ∥ [101]) showed slight nonlinearity below 50 K [Supplementary Figure 3(b)], but the other two were linear in field over the entire temperature range. The nonlinear data of S1 can be fit to the standard two-band model (Supp. Ref. [12])

$$\rho_{xy} = \frac{B}{e}\frac{(p\mu_p^2 - n\mu_n^2) + (p-n)(\mu_p\mu_n B)^2}{(p\mu_p + n\mu_n)^2 + (p-n)^2(\mu_p\mu_e B)^2} \tag{1}$$

with e the fundamental unit of charge, to extract the electron and hole densities (n and p, respectively) and carrier mobilities (μ_n and μ_p). Assuming no strong field dependence of the mobilities or carrier concentrations, the only way for ρ_{xy} to be nonlinear in this model is if the B^2 term is finite. Thus, $(p-n)$ must be nonzero, meaning that the material is not perfectly compensated. This single equation has three unknowns, so some assumptions must be made. We assume the relatively stable high temperature R_{H} values are dominated by the electron band signal, and calculate an approximate value $n = 5 \times 10^{21}$ cm^{-3}. A value of 2.5×10^{21} cm^{-3} is chosen for p, because the two carrier densities must be somewhat comparable to produce a nonlinear slope. The factor of two difference between n and p is not unreasonable, as roughly similar discrepancies have been seen in WP_2 (Supp. Ref. [13]) and WTe_2 (Supp. Ref. [14]), which have much more nonlinear ρ_{xy}. Values of p from 1-4 $\times 10^{21}$ cm^{-3} give equally good fits—in other words, the exact value is not crucial. The fit is more sensitive to the ratio of the mobilities, which we solve for. μ_n is always larger, unsurprising given the consistently negative R_{H}. But μ_n/μ_p drops dramatically from 50 K to 2 K [Supplementary Figure 3(b), inset; note the logarithmic y-axis], with values that are also robust to the choice of p; the hole mobility is becoming increasingly comparable to the electron mobility. A significant temperature dependence of the carrier mobilities is unsurprising when a material has a low resistivity (Supp. Ref. [3, 15, 16]).

The beginning of nonlinearity in the Hall effect for this orientation is close to T*, when angle-dependent MR becomes appreciable. The increasing presence of the hole band, due primarily to a change in mobility rather than carrier density, seems to contribute to the large magnetoresistance, as is the case for WTe_2 (Supp. Ref. [17]) and the cubic rare earth pnictides (Supp. Ref. [18]). However, the change in those materials is much more extreme, and ρ_{xy} actually changes sign with field. Even at 5 K in FeP, electrons are still eight times more mobile than holes, and the nonlinearity of the Hall resistivity is slight. The negative R_{H} at all orientations and temperatures is proof that electron transport is still dominant in FeP. While not as well compensated as the previously mentioned materials, or classic examples like bismuth (Supp. Ref. [12]), the electron and hole densities are close enough to lead to large, nonsaturating MR when the mobilities are also comparable. This, along with carrier concentrations orders of magnitude larger, is why the MR, while sizeable, does not reach the values of 10^5 or more seen with other compounds at similar field and temperature (Supp. Ref. [18–20]).

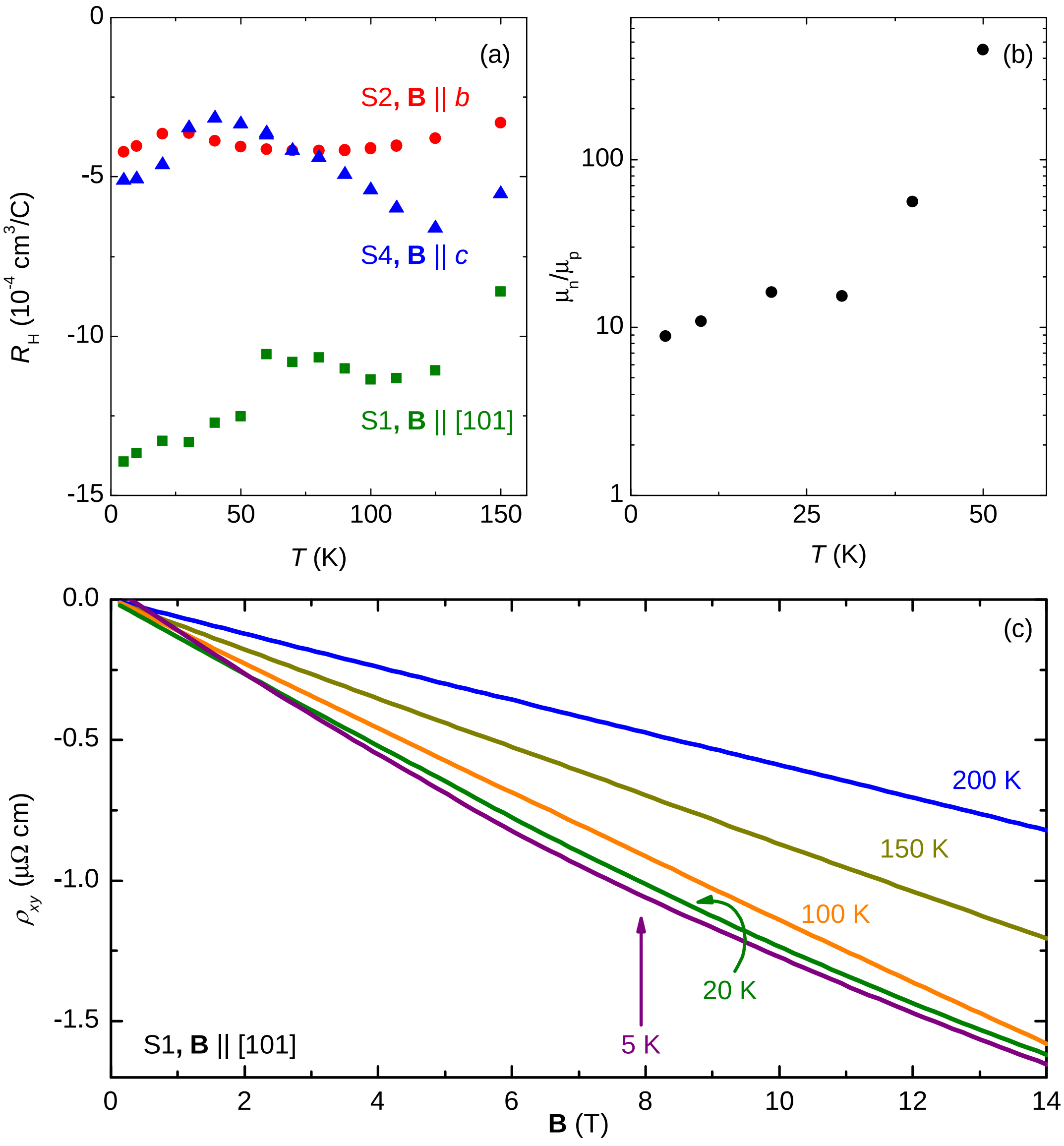

Supplementary Figure 3. (a) The Hall coefficient as a function of temperature for field along three different crystal orientations. (b) The ratio of the electron to hole carrier mobility, μ_n/μ_p, obtained from Eq. 1, at low temperatures where ρ_{xy} is no longer linear. (c) Hall resistivity at representative temperatures after antisymmetrization and smoothing of ±14 T field sweeps for **B** ∥ [101].

SUPPLEMENTARY NOTE 5. QUANTUM OSCILLATION TEMPERATURE DEPENDENCE

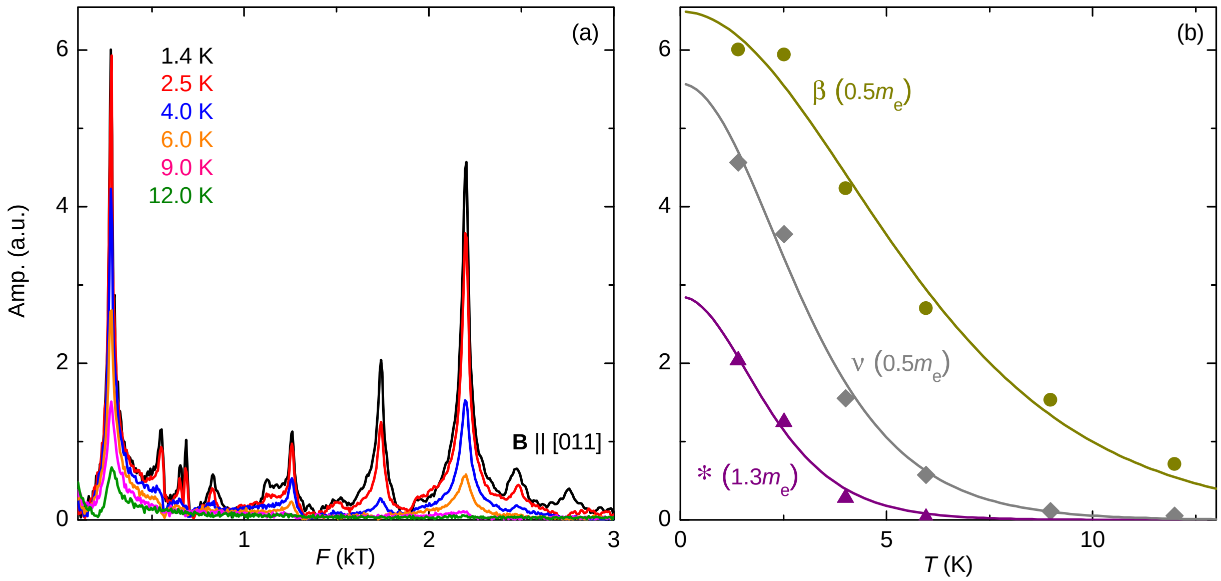

Supplementary Figure 4. (a) Fast Fourier transforms of the oscillatory torque signal for $\mathbf{B} \parallel$ [011] at several temperatures. (b) The amplitude of the β, ν, and previously unreported peaks (the last marked by an asterisk) in the FFT at different temperatures. Solid lines are fits to the expected Lifshitz-Kosevich temperature dependence, from which effective mass can be extracted.

The temperature dependence of the amplitude of quantum oscillations can be used to calculate the effective mass of individual oscillation frequencies. We did so for both the $\mathbf{B} \parallel$ [100] and $\mathbf{B} \parallel$ [011] directions for the sample presented in Fig. 6 of the main text. The expected temperature dependence of amplitude via the Lifshitz-Kosevich formula is $A = \frac{\alpha m^* T/(B m_e)}{sinh(\alpha m^* T/(B m_e))}$, where $\alpha = 2\pi^2 c k_B / e\hbar \approx 14.69$ T/K. Supplementary Figure 4(a) shows the FFTs from 1.4-12 K for $\mathbf{B} \parallel$ [011], while (b) shows the amplitudes of different peaks at various temperatures, with fits to the LK formula (solid lines). There is generally good agreement. Supplementary Table II lists the extracted effective masses for all the peaks whose amplitudes were large enough such that they were visible in the FFT to high enough temperature for an adequate fit. Those for field along the a-axis can be compared to the same orbits in Supp. Ref. [21]. We consistently found smaller effective masses than the previous work, with large differences in several cases. The explanation for this is not clear. They are also for the most part smaller than those predicted theoretically, though the masses derived from the SKEAF program are known to be less accurate than its predictions for the frequencies (Supp. Ref. [2, 22]).

Supplementary Table II. Effective masses extracted via the temperature dependence of the FFT peak amplitudes shown in Fig. 6 of the main text. They are compared to the SKEAF calculations and, for field along the a-axis, the data of Nozue et al. (Supp. Ref. [21]).

Label	$\mathbf{B} \parallel$ [hkl]	m^*/m_e (QO)	m^*/m_e (SKEAF)	m^*/m_e, Supp. Ref. [21]
γ	[100]	0.5	1.6	1.5
ι	[100]	0.5	1.0	2.3
κ	[100]	0.5	1.1	3.2
μ	[100]	2.1	1.8	2.9
β	[011]	0.5	4.6	—
2β	[011]	0.4	—	—
θ	[011]	0.4	4.1	—
ξ	[011]	0.4	3.3	—
λ	[011]	0.8	1.6	—
*	[011]	1.3	—	—
ν	[011]	0.5	1.6	—
π	[011]	0.5	1.5	—

SUPPLEMENTARY NOTE 6. ADDITIONAL C-AXIS DATA

Another FeP crystal with a much lower RRR than those shown in the main text (50, compared to typical values of 1000+) was also measured with high fields in aligned along the c-axis. As expected, the higher residual resistivity reduces the magnetoresistance (Supp. Ref. [12]), and in fact the sample resistance does not even double by 36 T. However, resistivity is still linear from low fields up to at least 24 K, though there is a clear non-linear region at lowest field. This is evidence that the linear MR phenomenon is independent from the large MR phenomenon, and that it does not require especially high quality samples.

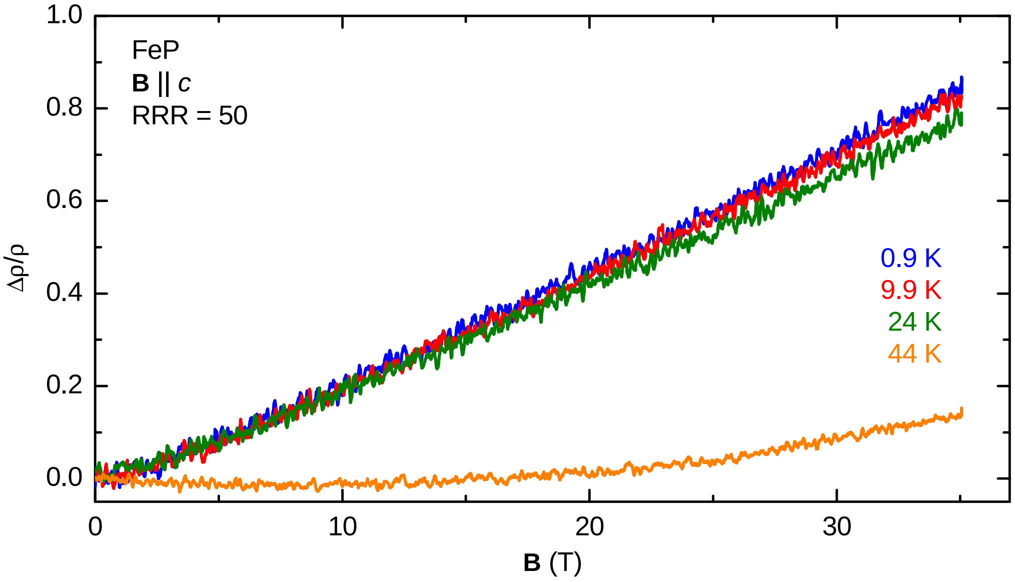

Supplementary Figure 5. High field magnetoresistance data with field applied along the c-axis for an FeP sample with a RRR of 50.

[1] Campbell, D. J. *et al.* CoAs: The line of 3*d* demarcation. *Phys. Rev. B* **97**, 174410 (2018).
[2] Campbell, D. J. *et al.* Quantum oscillations in the anomalous spin density wave state of FeAs. *Phys. Rev. B* **96**, 075120 (2017).
[3] Niu, Q. *et al.* Nonsaturating large magnetoresistance in the high carrier density nonsymmorphic metal CrP. *Phys. Rev. B* **99**, 125126 (2019).
[4] Niu, Q. *et al.* Quasilinear quantum magnetoresistance in pressure-induced nonsymmorphic superconductor chromium arsenide. *Nat. Comm.* **8**, 15358 (2017).
[5] Takase, A. & Kasuya, T. High field magnetoresistance in MnP. *J. Phys. Soc. Jpn.* **49**, 489–492 (1980).
[6] Du, J. *et al.* Large unsaturated positive and negative magnetoresistance in Weyl semimetal TaP. *Sci. China Phys. Mech.* **59**, 657406 (2016).
[7] Huang, X. *et al.* Observation of the chiral-anomaly-induced negative magnetoresistance in 3D Weyl Semimetal TaAs. *Phys. Rev. X* **5**, 031023 (2015).
[8] Shekhar, C. *et al.* Extremely large magnetoresistance and ultrahigh mobility in the topological Weyl semimetal candidate NbP. *Nat. Phys.* **11**, 645–649 (2015).
[9] Ghimire, N. J. *et al.* Magnetotransport of single crystalline NbAs. *J. Phys. Condens. Matter* **27**, 152201 (2015).
[10] Wang, Y.-Y. *et al.*. Resistivity plateau and extremely large magnetoresistance in $NbAs_2$ and $TaAs_2$. *Phys. Rev. B* **94**, 041103 (2016).
[11] Segawa, K. & Ando, Y. Magnetic and transport properties of FeAs single crystals. *J. Phys. Soc. Jpn.* **78**, 104720 (2009).
[12] Pippard, A. B. *Magnetoresistance in Metals*, vol. 2 (Cambridge University Press, 1989).
[13] Wang, A. *et al.* Large magnetoresistance in the type-II Weyl semimetal WP_2. *Phys. Rev. B* **96**, 121107 (2017).
[14] Luo, Y. *et al.* Hall effect in the extremely large magnetoresistance semimetal WTe_2. *Applied Physics Letters* **107**, 182411 (2015).
[15] Jo, N. H. *et al.* Extremely large magnetoresistance and Kohler's rule in $PdSn_4$: A complete study of thermodynamic, transport, and band-structure properties. *Phys. Rev. B* **96**, 165145 (2017).
[16] Wang, Y. L. *et al.* Origin of the turn-on temperature behavior in WTe_2. *Phys. Rev. B* **92**, 180402 (2015).
[17] Wang, Y., Wang, K., Reutt-Robey, J., Paglione, J. & Fuhrer, M. S. Breakdown of compensation and persistence of nonsaturating magnetoresistance in gated WTe_2 thin flakes. *Phys. Rev. B* **93**, 121108 (2016).
[18] Sun, S. *et al.*. Large magnetoresistance in LaBi: origin of field-induced resistivity upturn and plateau in compensated semimetals. *New J. Phys.* **18**, 082002 (2016).
[19] Tafti, F. *et al.*. Resistivity plateau and extreme magnetoresistance in LaSb. *Nat. Phys.* **12**, 272 (2016).
[20] Ali, M. N. *et al.* Correlation of crystal quality and extreme magnetoresistance of WTe_2. *Europhys. Lett.)* **110**, 67002 (2015).
[21] Nozue, T. *et al.*. de Haas-van Alphen effect of FeP in double helical magnetic state. *J. Phys. Soc. Japan* **70**, 192–198 (2001).
[22] Julian, S. Numerical extraction of de Haas–van Alphen frequencies from calculated band energies. *Comput. Phys. Commun.* **183**, 324–332 (2012).
